# Economic crisis and suicidal behaviour: the role of unemployment, sex and age in Andalusia, Southern Spain

**DOI:** 10.1186/1475-9276-13-55

**Published:** 2014-07-25

**Authors:** Juan Antonio Córdoba-Doña, Miguel San Sebastián, Antonio Escolar-Pujolar, Jesús Enrique Martínez-Faure, Per E Gustafsson

**Affiliations:** 1 Delegación Territorial de Igualdad, Salud y Políticas Sociales de Cádiz, Cádiz, Spain; 2 Department of Public Health and Clinical Medicine, Unit of Epidemiology and Global Health, Umeå University, Umeå, Sweden; 3 Empresa Pública de Emergencias Sanitarias, Cádiz, Spain; 4 Department of Public Health and Clinical Medicine, Unit of Family Medicine, Umeå University, Umeå, Sweden

**Keywords:** Suicide attempts, Economic crisis, Unemployment, Spain, Andalusia, Intento de suicidio, Crisis económica, Desempleo, España, Andalucía

## Abstract

**Introduction:**

Although suicide rates have increased in some European countries in relation to the current economic crisis and austerity policies, that trend has not been observed in Spain. This study examines the impact of the economic crisis on suicide attempts, the previously neglected endpoint of the suicidal process, and its relation to unemployment, age and sex.

**Methods:**

The study was carried out in Andalusia, the most populated region of Spain, and which has a high level of unemployment. Information on suicide attempts attended by emergency services was extracted from the Health Emergencies Public Enterprise Information System (SIEPES). Suicide attempts occurring between 2003 and 2012 were included, in order to cover five years prior to the crisis (2003–2007) and five years after its onset (2008–2012). Information was retrieved from 24,380 cases (11,494 men and 12,886 women) on sex, age, address, and type of attention provided. Age-adjusted suicide attempt rates were calculated. Excess numbers of attempts from 2008 to 2012 were estimated for each sex using historical trends of the five previous years, through time regression models using negative binomial regression analysis. To assess the association between unemployment and suicide attempts rates, linear regression models with fixed effects were performed.

**Results:**

A sharp increase in suicide attempt rates in Andalusia was detected after the onset of the crisis, both in men and in women. Adults aged 35 to 54 years were the most affected in both sexes. Suicide attempt rates were associated with unemployment rates in men, accounting for almost half of the cases during the five initial years of the crisis. Women were also affected during the recession period but this association could not be specifically attributed to unemployment.

**Conclusions:**

This study enhances our understanding of the potential effects of the economic crisis on the rapidly increasing suicide attempt rates in women and men, and the association of unemployment with growing suicidal behaviour in men. Research on the suicide effects of the economic crisis may need to take into account earlier stages of the suicidal process, and that this effect may differ by age and sex.

## Abstract (es)

**Resumen (Spanish/Español):**

**Introducción**

A pesar de que las tasas de suicidio han aumentado en algunos países europeos en relación con la crisis económica actual y las políticas de austeridad, esa tendencia no se ha observado en España. Este trabajo examina el impacto de la crisis económica sobre los intentos de suicidio, punto final del proceso suicida poco estudiado hasta ahora, y su relación con desempleo, edad y sexo.

**Métodos**

El estudio fue llevado a cabo en Andalucía, la región más poblada de España, y con un alto nivel de desempleo. La información sobre los intentos de suicidio atendidos por los servicios de emergencia se obtuvo del Sistema de Información de la Empresa Pública de Emergencias de Salud (SIEPES). Se incluyeron los intentos de suicidio ocurridos entre 2003 y 2012, con el fin de cubrir los cinco años anteriores a la crisis (2003–2007) y cinco años desde su inicio (2008–2012). Se recuperó información de 24 380 casos (11 494 hombres y 12 886 mujeres) sobre sexo, edad, dirección y tipo de atención recibida. Se calcularon las tasas de intentos de suicidio ajustadas por edad. Se estimó el exceso de intentos de suicidio en 2008–2012 para cada sexo utilizando las tendencias históricas de los cinco años anteriores con modelos de regresión temporal mediante regresión binomial negativa. Para evaluar la asociación entre el desempleo y las tasas de intentos de suicidio se ajustaron modelos de regresión lineal con efectos fijos.

**Resultados**

Se detectó un importante aumento de las tasas de intento de suicidio en Andalucía tras el comienzo de la crisis, tanto en hombres como en mujeres. Los adultos de 35 a 54 años fueron los más afectados en ambos sexos. Los intentos de suicidio se asociaron con el nivel de desempleo en los hombres, explicando casi la mitad de los casos durante los cinco primeros años de la crisis. Las mujeres también se vieron afectadas durante el período de recesión, pero el incremento de los intentos de suicidio no pudo atribuirse específicamente al desempleo.

**Conclusiones**

Este estudio mejora nuestra comprensión de los efectos potenciales de la crisis económica sobre el rápido aumento de los intentos de suicidio en mujeres y hombres, y de la asociación del desempleo con el auge de la conducta suicida en los hombres. La investigación del impacto de la crisis económica sobre el suicidio debe tener en cuenta las etapas iniciales del proceso suicida, y que sus efectos pueden manifestarse de forma diferente según edad y sexo.

## 

There is an emerging concern over the effects on health of the economic recession that started in 2008 [1]. Despite the observation that some indicators may improve during crises, a diversity of mortality and morbidity outcomes has shown to be affected [2]. One central example of this phenomenon are the increased suicide rates in several European countries, which are believed to be triggered by the current economic downturn and the austerity policies implemented by national governments [3,4]. Dramatic reductions in social expenses are contributing to a restricted access to social services and benefits for the most vulnerable population groups, in some cases under the umbrella of financial adjustment promoted by the Troika (comprising the European Central Bank, International Monetary Fund and the European Commission) [5].

Despite the fact that Spain is one of the countries most severely affected by unemployment and austerity policies during the present economic crisis [6], suicide rates have remained almost unchanged at relatively low levels during the period [7]. The same picture is observed in Andalusia, the most populated region of Spain, with very high unemployment rates, where suicide rates has been declining steadily for the previous years in both sexes [8].

One possible clue to this apparent paradox is that too little attention has yet been paid during the present crisis to the different stages of the suicidal process. The suicidal process framework classifies suicidal behaviour starting with suicidal ideas and thoughts, progressing to plans, then growing through suicidal attempts and finally to fatal suicide [9].

Suicide attempts, not commonly included in official statistics, have proved to be meaningful in evaluating mental health in various settings [10]. Thus, this approach can be very useful considering that suicide underreporting has been shown to be high worldwide, Spain included [11]. Previous research has highlighted that serious suicide attempters and suicide victims can be regarded as overlapping populations sharing common characteristics [12]. For example, a prior suicide attempt is the strongest risk factor for completed suicide. Attempted suicide increases the risk of mortality several years later [13] and in the long term, up to 12% of attempters eventually complete suicide [14].

With regard to suicide attempts, recent studies have shown that the current economic downturn is associated with an increase in suicide attempts in Ireland [15] and also with rising prevalence of suicidal thoughts in Greece [16].

Economic crises may affect suicide attempts incidence in different ways, either strengthening risk factors, or weakening protective factors. In the first case, crises contribute to increasing unemployment, poverty, financial problems and social deprivation. In the second pathway, economic recessions frequently entail retrenchments in job security and cuts in welfare protection programs, such as unemployment benefits [17]. Unemployment has for long been used as the natural indicator of an economic crisis, both at the individual and ecological level [18]. In addition, there is an unambiguous relationship between unemployment and suicide [19] and suicide attempts [20] also in non-crisis settings.

Another issue of potential importance is that some studies suggest that unemployment and other socio-economic variables have greater effects on suicide in men than in women [21]. Diverse mechanisms have been suggested to explain this gender difference in the effect of unemployment, besides alteration in socio-economic position, especially in terms of loss of status, routine and social support [22]. The persistence of traditional family roles could also be associated with sex-differentiated suicide behaviours. While several studies have measured the association between unemployment increase and suicide during the current recession [23-25], only one of them was stratified by sex [26]. This ecological study found that suicide increased in men more than in women in the first years of the crisis, though this association was only detectable in countries with low pre-crisis unemployment rates. Based on this meagre literature, a differential effect of unemployment by gender is possible during economic crisis, but may not be expected in a setting with high levels of unemployment before the crisis, such as Andalusia.

In a similar way as for gender, it is possible that the association between unemployment and suicidal behaviour is affected by age in the downturns [27]. This topic has not been pointed out in the Spanish research in relation to the current crisis, except in specific studies at the local level [28].

In summary, suicide mortality trends have so far not revealed any negative impact in relation to the current economic crisis in Andalusia. It is possible that the lack of information on previous stages of the suicidal process, such as suicide attempts, could be concealing detrimental effects. Moreover, the potential differential effects by sex and age of the crisis on suicide attempts has been insufficiently studied. The aim of this paper is to examine the trends in suicide attempts in Andalusia during the economic crisis and in relation to high unemployment rates. A second aim is to explore possible sex and age differences in this relationship.

## Methods

### Setting

Andalusia is the largest and most populated (8.45 million, density 96/km^2^) region in Spain. Placed in the south of the country, it is divided into 8 provinces. Although it has overcome much of its historical lag in recent decades, many of its social and economic indicators are still below the Spanish average. Per capita GDP was 16.960€ in 2012. Unemployment rose in Andalusia from 12.2% in 2006 to 35.8% in 2012 [29] and poverty rates increased from 29.5% in 2008 to 31.0% in 2012, far above the Spanish poverty rate of 22.2% [30].

The Spanish health system is essentially decentralised and each one of the 17 autonomous regions, including Andalusia, has a high level of autonomy in the planning and provision of health services. Only general policies such as foreign health affairs and legislation on medicinal products and medical devices are established at the central level [31].

Until 2012, health coverage in Andalusia, including emergencies and mental health services, was guaranteed for all the population. The total service provision in primary health care and emergencies was publicly managed and only 5% of hospital services are publicly funded but with a private provision. Until this date, there were no user fees, and co-payment was required only at ambulatory pharmacies (with exemptions for the elderly and the unemployed). Mental health care in Andalusia, on a universal coverage basis, is provided by the public health system. It is integrated with the primary care network, and the specialised and emergency networks. Mental health services attend acute, middle and long mental therapies in a variety of facilities.

Emergencies in Andalusia are attended at primary health care centres, at hospital emergency wards, or through mobile units. The public enterprise of health emergencies (EPES by its initials in Spanish) has a provincial level of organisation, and is in charge of coordinating the mobile units in case of life-threatening pre-hospital cases.

### Sample

Information on suicide attempts was extracted from the Health Emergencies Public Enterprise Information System (SIEPES). In Andalusia, patients (or families) in need of acute or life-threatening pre-hospital emergency assistance can dial the 112 or 061 phone numbers. All health emergency calls are channelled to and managed at a Health Emergency Coordination Centre, one for each of the eight provinces of Andalusia. Depending on the severity and location of the event, either basic life support ambulance or mobile intensive care units are sent to attend the emergency situation [32]. With the help of a specific computerized decision support, the information is entered into the computer in a structured manner by trained non-clinical call takers. According to the reason for the call, a specific algorithm is followed, and the information of the responses to various closed-ended questions is recorded. In our case, whenever the the word “suicide” appears in some of the responses’ options, an X84 code is automatically generated. The call takers triage calls and have access to a medical advisor when necessary, always before deciding the allocation of any type of mobile unit and team to attend an emergency. Only exceptionally a physician is not present in the emergency mobile unit team attending a suicide attempt case. This procedure is identical for the eight province Coordination Centres.

This system has been expanding since 1990 and covered all the population homogeneously, both in urban and rural areas about ten years before the study period.

In this study, all cases from SIEPES registry aged 15 to 64 and with the suicide attempt code, between January 1^st^ 2003 and December 31^st^ 2012 were included, in order to cover five years prior to the crisis (2003–2007) and five years since it started (2008–2012). Information on sex, age, address, and type of attention provided was also retrieved from SIEPES. This information is collected initially by the call taker if available, and in some cases it is completed by the emergency care team during medical assistance.

This source of information is independent from alternative sources of data on suicidal behaviour like hospitalised suicidal attempts, which are registered in hospital discharge data systems, or completed suicide data, which are extracted from death certificates.

Unemployment data were obtained from the Active Population Survey of the National Institute of Statistics, performed quarterly [29].

Between 2003 and 2012, there were 32,468 calls coded as suicide attempts. Of them, 27,963 occurred in patients aged between 15 and 64. After discarding 1,859 for later cancellation by the user and 1,036 for absence of the patient when the mobile unit arrived, 25,068 remained. Finally, 688 cases were excluded because the sex coding was missing, resulting in 24,380 cases: 11,494 men and 12,886 women. In relative terms, the demands for attention for suicide attempts accounted for 0.20% of all telephone calls to the EPES emergency service in 2003 and for 0.47% in 2012.

### Analysis

Episodes of suicide attempt were classified according to sex, age, year and province of residence. European population adjusted rates (per 10^5^ population) were calculated for each year and province using population data from the Andalusian Institute of Statistics and Cartography. We considered that the current recession started at the end of 2007, so in our study the pre-crisis period ranged from 2003 to 2007, and the crisis period from 2008 to 2012.

To evaluate possible change in rates, excess numbers of attempts from 2008 to 2012 were calculated for each sex using the historical trends of the five previous years through time regression models using negative binomial regression analysis.

To assess the association between unemployment (percentage of unemployed) and suicide attempt rates, linear regression models with fixed effects were performed, to remove potential confounding at the province level [25], and were stratified by sex. In a second step, we included dummy variables for year in order to control for temporal trends. In both cases, robust estimations were calculated.

In the results from the first model (without year adjustment), the coefficient of unemployment rates can be interpreted as a general nonspecific indicator of the effects of the crisis on suicide attempts, and in the second model, adjusting for time trends, the estimate can be interpreted as specific effects of the unemployment rates.

Finally, we carried out separate analyses for each five-year age groups specific attempt rates including global unemployment rates, for province and year, as the independent variable, always adjusting for year, and stratifying by sex.

All analyses were performed using the Stata 12 software.

## Results

### Suicide attempts by year, sex and age

**Figure 1:**
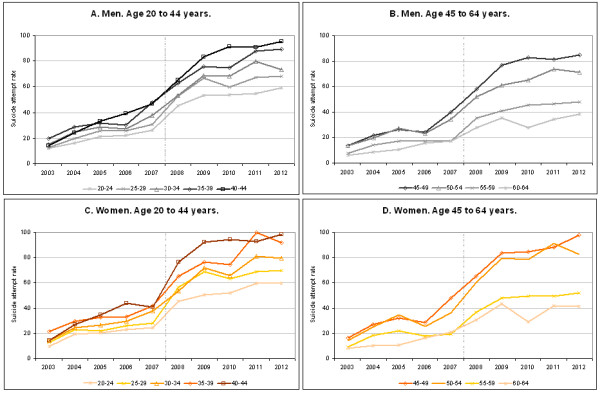
**Changes in suicide attempts rates (× 10**^**5**^**) according to age groups and sex, 2003–2012. Panel A**: Men from 20 to 44 years. **Panel B**: Men from 45 to 64 years. **Panel C**: Women from 20 to 44 years. **Panel D**: Women from 45 to 64 years. Vertical dash-dotted lines indicate the onset of recession.

**Figure 2:**
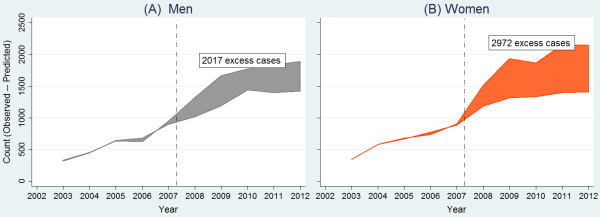
**Time trend analysis of excess numbers of suicide attempts in Andalusia between 2008 and 2012 in men (A) and in women (B).** Vertical dash-dotted lines indicate the onset of recession.

The number of cases and annual adjusted rates of suicide attempts for Andalusian men and women between 15 to 64 years are shown in Table 1. Women had higher rates during the entire period. A slightly decreasing trend in annual relative differences by sex was shown, going from above 1.4 in the first years to around 1.2 at the end of the period.Suicide attempts rates according to age are shown in Figure 1. There was an age gradient during the whole period for both men and women, with consistently higher rates in the 40 to 44 age group, and lower rates in the 20 to 24 group. Graphically, there was a clear increasing trend in the age gap in rates, starting in 2008. From age 45, the trends in suicide attempts decreased; that is, the 45 to 49 age group showed lower rates than the 40 to 44 group, and the rest of the five-year age groups rates decreased gradually from there.Between 2008 and 2012 there was an excess of 4,989 (95% CI: 1,985-8,013) suicide attempts, 2,017 (95% CI: 87–3,987) in men and 2,972 (95% CI: 1,878-4,075) in women, i.e. compared to if the historical trends prior to the onset of the crisis had been stable (see Figure 2).

**Table 1:** **Age adjusted suicide attempt rates (×10**^**5**^**) and unemployment rates in Andalusian population by sex between 2003 and 2012**

	**Suicide attempts**						**Unemployment rates**	
	**Men 15 to 64**		**Women 15 to 64**		**Rate ratio women/men**	**95% CI**	**Men 15 to 64**	**Women 15 to 64**
	**n**	**Adjusted rate × 10**^**5**^	**n**	**Adjusted rate × 10**^**5**^				
2003	330	13.1	344	19.1	1.46	1.25 - 1.69	13.72	26.34
2004	459	17.5	585	28.0	1.60	1.42 - 1.81	12.58	24.15
2005	636	23.1	684	31.4	1.36	1.22 - 1.52	10.27	19.45
2006	628	22.7	737	32.5	1.43	1.28 - 1.59	9.23	17.94
2007	949	33.2	908	38.4	1.16	1.06 - 1.27	9.48	17.61
2008	1323	45.7	1520	59.1	1.29	1.20 - 1.39	15.23	21.46
2009	1666	56.5	1937	73.0	1.29	1.21 - 1.38	24.08	27.09
2010	1775	59.9	1868	70.1	1.17	1.10 - 1.25	26.87	29.41
2011	1837	61.6	2154	79.5	1.29	1.21 - 1.37	28.88	32.33
2012	1891	63.7	2149	79.0	1.24	1.17 - 1.32	33.58	35.84
Total	11494		12886					

### Unemployment and suicide attempts

The fixed effects regression models indicated that between 2003 and 2012 each annual 1% increase in unemployment rate was associated with a rise of 1.81 units (95% CI: 1.51-2.11) in the rate of suicide attempts (per 10^5^) in men and 2.27 (95% CI: 1.55-2.99) in women (Table 2).

**Table 2:** **Linear regression fixed effects models for suicide attempt rates (×10**^**5**^**) regressed on unemployment rates (%) in Andalusia for men and women**

**Men**	**Model 1**				**Model 2**		
		**Coef**	**p**	**95% CI**	**Coef**	**p**	**95% CI**
Constant		3.79	0.15	-1.69 - 9.27	-2.05	0.78	-18.47 - 14.3
Unemployment (%)		1.81	<0.01	1.51 - 2.11	1.08	0.04	0.06 - 2.09
Year	2003				ref	-	-
	2004				4.63	0.121	-1.58 - 10,85
	2005				11.60	0.002	5.78 - 17,13
	2006				13.21	0.002	6.76 - 19.66
	2007				22.05	0.001	13.20 - 30.90
	2008				27.89	0.000	20.10 - 35.67
	2009				29.66	0.000	20.30 - 39.03
	2010				29.29	0.001	17.72 - 40.86
	2011				27.56	0.007	10.41 - 44,72
	2012				25.68	0.034	2.59 - 48.77
**Women**	**Model 1**				**Model 2**		
		**Coef**	**P**	**95% CI**	**Coef**	**p**	**95% CI**
Constant		-11.3	0.19	-29.6 - 7.03	5.46	0.80	-43.98 - 54.8
Unemployment (%)		2.27	<0.01	1.55 - 2.99	0.49	0.52	-1.23 - 2.21
Year	2003				ref	-	-
	2004				8.63	0.071	-0.98 - 18.24
	2005				13.39	0.087	-2.55 - 29.32
	2006				14.16	0.081	-2.27 - 30.59
	2007				20.00	0.033	2.16 - 39.19
	2008				37.45	0.001	20.95 - 53.94
	2009				48.51	0.000	36.13 - 60.89
	2010				43.93	0.000	29.73 - 58.14
	2011				50.73	0.000	34.68 - 66.77
	2012				49.79	0.001	29.84 - 69.74

Model 1 = crude model; Model 2 = adjustment for yearly trends.

In the second fixed effects model we included a dummy variable for the year to further adjust for time trends. This model showed that 1% increase in unemployment was related to an increase of 1.08 units (95% CI: 0.06-2.09) in suicide attempt rate in men, and to a non-significant rise of 0.49 units (95% CI: -1.23-2.21) in women (Table 2).

Taking into consideration the changes related to unemployment rates variations, we estimated the number of cases of suicide attempts potentially associated with unemployment. Average unemployment rate for men in the second period was 25.7%. Following our Model 2, this yielded an associated attempt rate of 27.8 × 10^5^ (that is, 25.7 times 1.08, the coefficient for unemployment). Taking into consideration the average population in the period it gave an estimated number of cases of 4,101 (95% CI: 228–7,935). Unemployment thus accounted for 48.3% of the total 8,492 suicide attempt cases in the five initial years of the downturn (2008–2012).

The separate analyses for age specific attempt rates and global unemployment rates showed that in men the stronger associations, though not consistently significant, were found in 40–54 age groups. The 20–24 year group in men and 30–34 group in women also presented significant coefficients (Table 3).

**Table 3:** **Linear regression fixed effects models for age specific suicide attempt rates (×10**^**5**^**) regressed on unemployment rates (%) in Andalusia for men and women, 2003-2012**

	**Men**			**Women**		
**Age group**	**Coef**	**p**	**95% CI**	**Coef**	**p**	**95% CI**
20-24	1.91	0.010	0.63 - 3.19	0.68	0.469	-1.41 - 2.77
25-29	0.32	0.754	-2.03 – 2.68	0.53	0.522	-1.33 - 2.39
30-34	0.84	0.390	-1.32 - 2.99	1.05	0.046	0.00 - 2.06
35-39	0.76	0.502	-1.79 - 3.32	0.09	0.944	-2.75 - 2.92
40-44	1.48	0.152	-0.70 - 3.65	0.46	0.745	-2.77 - 3.70
45-49	1.41	0.233	-1.15 - 3.97	0.24	0.764	-1.60 - 2.09
50-54	1,68	0.022	0.32 - 3.03	1.01	0.441	-1.91 - 3.92
55-59	0.70	0.476	-1.50 - 2.89	0.33	0.771	-2.24 - 2.90
60-64	0.32	0.560	-0.92 - 1.57	0.15	0.903	-2.66 - 2.96

Models adjusted for yearly trends.

## Discussion

In contrast to statistics showing a perplexing decrease in suicide during the first years of the economic crisis in Spain, the present study detected a sharp increase in suicide attempt rates in Andalusia, both in men and in women. The age groups 35 to 54 were the most affected. Moreover, suicide attempt rates were associated with unemployment rates in men, accounting for almost half of the cases during the five initial years of the crisis. In contrast, the substantial increase in suicide attempts in women could not be specifically attributed to unemployment.

The findings of the current study are consistent with some previous research on the impact of the economic crisis on different stages of the suicidal process. Perry et al. described a change to increasing rates after 2006 in the Irish population deliberate self-harm registry. Moreover, they highlighted successive annual 10% increases in suicide attempt rates in men during 2008 and 2009 [15]. In a similar vein, a hospital-based study in northern Spain detected a two-fold increase in suicide attempt rates concurring with the early years of the economic downturn [33]. That is a smaller increase than ours, maybe due to higher initial rates in the referred study.

A recent article by Chang et al. [26] approaching the impact of global crisis on suicide rates in 54 countries worldwide found a greater increase in suicide in men than in women in countries with a low starting unemployment rate but not in countries with a pre-crisis unemployment rate higher than the median (6.2%). In contrast to these findings, however, we detected this association in a setting with very high levels of structural unemployment (>10% in the pre-crisis period).

Our results support previous evidence that socio-economic factors are more strongly associated with suicide [21] and suicidal behaviour [34] in men than in women, in the present study represented by unemployment rates. One possible explanation to this finding is that in Andalusia the working role has remained an essential constituent of masculinity. Men are submitted to a more pronounced pressure for their status as breadwinners, and therefore unemployment and uncertainty about future employment may have a stronger impact on their health than on women’s, who can better compensate because of their traditional family roles [35]. Assumption of traditional gender roles, still prevalent in the setting of the present study, makes men more dependent on relative socio-economic success and control over their work and, therefore, more sensitive to deprivation, and as a consequence, of unmet expectations [36], also in relation to social class [37]. This role adherence may contribute indirectly to increased suicide risk by exercising a negative influence on mental states, social support and help-seeking behaviour [38].

The analysis of age rates indicated that the middle-aged population, between 35–44 years, are the most affected group. This result is in accordance with another study performed in northern Spain during the ongoing crisis [28]. Moreover, recent research described an increase in prevalence of poor mental health in Spanish men aged 35 to 54, especially breadwinners, attributable to unemployment during the initial years of current recession [39]. Similar to the gender effects, these findings could be attributed to the pressure these groups received to be the main earners. Since mid-adulthood is a period in life when the financial burden of the family and young children may be marked, and during which one may not be established in the labour market, the threat of unemployment is the greatest. Bankruptcy of small businesses, evictions and mortgage repayment difficulties have been associated with an increase in the risk of depression in Spain [40] and it is the people in this age range who are frequently confronted with these financial crisis-related events.

One unanticipated finding of our study was the strong association detected between specific suicide attempt rates in men 20–24 year and global unemployment rates. We consider that difficulties in entering the labour market for young adults may play an important role in this association. Unemployment rate in men under 24 was above 50% in our setting during the study period. Previous research suggests that besides financial problems, pessimism about the future and high demands on this group are in the pathway to poor mental health in times of economic crisis [41].

### Methodological considerations

As far as we know, this is the first ad-hoc study of the impact of the current economic crisis on suicide attempts based on emergency service registries in a wide population setting. Mobile emergency services information has not frequently been used as a source of mental health information due to lack of coverage or sensitivity. In this case, the methodology appears to be sensitive to changes in mental health that probably remain undetected by the suicide mortality official data [11]. The only deliberate self-harm registry so far recognised is in Ireland, and gathers information on 40 hospitals’ emergency wards country-wide [15]. The apparent contradiction between the clear upward trend in suicide attempt rates and the steady suicide mortality rates in Andalusia during the beginning of the current economic crisis could be attributable, firstly, to the underreporting of suicide data, as previously stated. Secondly, we could also consider a plausible time lag between the onset of the crisis and mortality outcomes which might differ from that of suicide attempts; as long as welfare protection programs and services such as public health provision still are functioning, they might have a buffering effect on serious mental health consequences of the crisis. Preliminary official mortality data indicate a rise in suicide cases in Spain during 2012, though not consistent in all autonomous regions as Andalusia. We consider that evidence from published studies to date indicates that our results are in accordance to the suicide attempt rates increases detected in several Northern Spanish regions [28,33] which suggests our findings can be generalized to the entire country, with necessary caution due to methodological specificities.

Our study has the limitations of an ecological design when studying the association of unemployment with suicide attempt rates. The analyses followed the methodology of several recent investigations performed on suicide mortality and unemployment in the early years of the current crisis in other European countries [23,24] and the US [25], though none of them explored sex differences regarding unemployment. It should be emphasized that there are a number of potential pathways other than unemployment by which the crisis can impact on suicide attempts such as through GDP. Complementary analyses including GDP in the model (data not shown), did however not find any significant association between GDP and suicide attempts (p = 0.32 in men and 0.76 in women). Nevertheless, other pathways not considered in this paper, such as unemployment benefits, may be of importance.

A second limitation of our research is that even though public emergency health service covers all the population with no limits for access, a proportion of patients is firstly attended at hospital or primary care emergency units and thus not included in our sample. However, no relevant changes in health service use has been detected in the period of study and the use of EPES services has increased steadily during the period, with the suicide attempts share increased more rapidly (data not shown).

### Implications for health policy

These findings may help us to understand the need to foster social policies that are addressed to both the unemployed and the general population. As previous research has established, when the social security system is more comprehensive, unemployment is less likely to affect mental illness and suicide [42]. At the moment, these policies are far from the Spanish government austerity agenda, which has proved to be ineffective to date [5].

Our study also suggests the role of primary care teams in preventing suicide by detecting suicidal thoughts in high-risk groups such as the unemployed, and providing patients with the appropriate counselling, treatment or referral [43]. There is a challenge to increase access to primary care and mental health services for unemployed men.

The usefulness of universal coverage mobile emergency services data on mental health needs to be further tested in relation to hospital- and population-based registries. Further research is also required on factors other than unemployment, such as evictions, that could be influencing the effect of the current recession on people’s health.

## Conclusion

The current findings add substantially to our understanding of the impact of economic crisis on the rapidly increasing suicide attempt rates in women and men, and the association of rising unemployment rates with suicidal behaviour in men. The study suggests that future research on the suicide effects of the economic recessions should take into account earlier stages of the suicidal process than completed suicide, and also that this effect may differ by age and sex.

## Competing interests

The authors declare that they have no competing interests.

## Authors’ contributions

JAC and AEP conceived the original study. AEP and JMF supervised all data collection. JAC performed the literature review and statistical analyses and wrote the article. MSS, AEP and PEG provided consultation regarding conceptualization, analysis and interpretation of findings. JAC, MSS, AEP, JMF and PEG contributed to the article by reviewing the manuscripts. All authors have read and approved of the final version.
